# The Proctologist

**Published:** 1900-05-26

**Authors:** 


### THE PROCTOLOGIST.

It is interesting to note the relation between the development of specialism and the invention of special instruments of research, by aid of which powers of diagnosis beyond those possessed by ordinary practitioners have been placed at the disposal of those who have familiarised themselves with their use. As examples, we may point to the ophthalmoscope, the laryngoscope, and the cystoscope, the use of each of which in its early days did much to make into a specialty the maladies with which it was concerned; and now we have the proctoscope and the proctologist. In America they like to have names for things, and these are the names they give to the instrument they use for examining the rectum, and the gentleman who practises that delightful specialty. Of course, we have the specialty in England in pretty full bloom, but we do not think that as yet its professors claim the appellation of proctologists. In America, however, they have an "American Proctologic Society," they have "Professors of Proctology" in their medical schools, and they have "Proctologists" to their hospitals; and, as is the case in every rapidly growing specialty, the younger votaries of what we suppose will soon be called a science do not hesitate to throw mighty scorn upon the ancient methods of "ten years ago." There is however, a good deal in proctology; there can hardly be a doubt, for example, that "obstipation," or the difficulty arising from hypertrophy of the rectal valve, is a real and troublesome condition; and it seems very probable that in some cases relief from it can be obtained by the operation which goes by the name of "valvotomy," and, of course, in regard to ulcers much is gained by the "new method" and the "new posture." To the proctologist position is everything, and, in fact, much of the technique of the specialty hinges on the fact that if a patient is placed in such a position that the abdominal walls fall away from the spine, and so by their weight produce a minus pressure in the rectum, and if then air be admitted to this cavity, "ballooning" takes place, and this to such an extent that the whole of the mucous membrane can be inspected by aid of a properly devised instrument. By this manœuvre it is held to be possible to see quite into the lower portion of the sigmoid, that part which not so many years ago was held to be the dark spot to which we could reach neither from below or from above. On this is founded proctology and the proctologist.

